# Supplementary material for: Are carbon emissions trading and green financial instruments synergistic? -Comprehensive quantitative research based on content analysis
Source: PLoS One. 2024 Mar 7;19(3):e0298601. doi: 10.1371/journal.pone.0298601 (PMC10919601; doi:10.1371/journal.pone.0298601)
Supplement: S1 Data — (ZIP) [file pone.0298601.s001.zip › Data/地级市Z_F工作报告.zip!/║╙▒▒/║¬╡a╩╨/└┤╫╘╣1⁄2╓┌║┼í╛┬φ┐╦╩2╛▌═°í┐_2017.docx]

各位代表：

现在，我代表市人民政府向大会作工作报告，请予审议，并请市政协委员和列席会议的同志提出意见。

一、过去四年工作回顾

市十四届人大一次会议以来的四年，是邯郸发展史上极不平凡的四年。全市人民紧密团结在以习近平同志为核心的党中央周围，在省委、省政府和中共邯郸市委的坚强领导下，深入贯彻落实习近平总书记系列重要讲话精神，牢固树立五大新发展理念，主动适应经济发展新常态，着力推进供给侧结构性改革，围绕建设宜居宜业宜游富强邯郸、美丽邯郸的战略目标，开拓创新，拼搏奋进，“十二五”规划顺利实施，“十三五”规划开局良好，较好完成了各项工作任务。

（一）全力稳定经济增长，综合实力不断提升。认真落实中央、省各项稳增长政策，坚持“稳中快进”工作总基调，有效应对下行压力，精准出台了一系列创新性措施，持续增加有效投资，扩大消费需求，推动经济发展迎来由慢到快的重要拐点。特别是连续开展10轮项目建设“三集中”活动，集中签约项目657个，集中开工项目1479个，武安新能源产业园、五粮液北方灌装基地、中材风电叶片、中船重工汉光光电产业园、华耀城等一批重大产业项目顺利实施，发展后劲显著增强。2016年，全市生产总值达3337.1亿元，四年年均增长6.8%；一般公共预算收入204.5亿元，年均可比增长3.9%；规模以上工业增加值1182.5亿元，年均增长5.7%；固定资产投资3765.2亿元，年均增长13.2%；社会消费品零售总额1508.9亿元，年均增长11.5%。

（二）持续调整产业结构，转型升级步伐加快。三次产业结构由2012年的12.7: 53.6: 33.7，调整优化为12.5:47.2 :40.3。全面落实“三去一降一补”重点任务，累计压减炼铁产能1148万吨、炼钢产能1004万吨、煤炭产能276万吨、焦炭产能553万吨、水泥产能464万吨。传统产业加快改造，新兴产业发展迅速，装备制造业、高新技术产业增加值占规模以上工业比重分别达16.6%和14.5%，比2012年提高8.2个和8.6个百分点，六大高耗能行业增加值占比下降10.7个百分点，荣获国家循环经济试点示范城市。规模以上工业企业达1350家，四年净增419家。服务业全面升级，商贸物流、电子商务迅速发展，市区南部现代商贸物流隆起带初具规模，娲皇宫、广府古城被评为5A级景区，旅游业总收入年均增长37%。现代农业稳步发展，粮食总产稳定在105亿斤以上，建成省级以上农业科技园区12家、省级现代农业园区13家，省级以上重点龙头企业达75家、居全省第一，农业产业化经营率达67%、比2012年提高4.7个百分点。

（三）积极推动协同发展，开放水平日益提高。抢抓京津冀协同发展战略机遇，加大对接央企力度，累计与新兴际华、中国建材等央企签订124个项目合作协议。主动承接京津产业转移，打造重点承接平台，邯郸经济技术开发区晋升为国家级开发区，冀南新区荣获国家级产城融合示范区、省级高新技术产业开发区，冀津循环经济产业示范区获批成立，省级以上经济开发区达20家，建成曲周自行车、广平家具、邱县休闲食品等一批京津产业转移基地。致力打造协同创新共同体，成立了北京大学邯郸创新研究院，教育、医疗、交通、旅游等多领域合作成果丰硕，大气污染联防联控取得积极成效。对外开放不断拓展，白俄罗斯戈梅利、德国优布劳、珠海银隆、深圳华强、星星集团等一批国内外知名企业落户邯郸。借力“一带一路”战略，推动优势产能“走出去”，文安马来西亚钢厂项目签约。全市实际利用外资累计完成36.5亿美元，出口总值累计完成398.2亿元。

（四）全面深化改革创新，发展活力明显增强。深入推进“放管服”改革，下放225项行政审批事项，非行政许可审批事项全部取消，整合建立了统一的公共资源交易平台，组建了市行政审批局，行政审批规范化标准化经验在全省推广。市县政府机构改革、市级机关内设机构改革、不动产统一登记机构整合等改革任务全部完成。财税金融改革不断深化，“营改增”全面推开，国地税实行联合办税，挂牌上市企业101家，驻邯商业银行18家，邯郸银行保定分行建成开业。2016年末，全市各项贷款余额3129.3亿元，比2012年末增长70%。107家国有企业实施改制破产重组。农村土地承包经营权确权登记面积完成95%。实施市民网、市民车、市民卡“三大市民工程”，推出小学“弹性离校”、“借道左转”、公益直饮水等措施，便民利民改革深受群众好评。科技创新步伐加快，国家高新技术企业达210家，科技型中小企业6061家，院士工作站20家，市级以上众创空间29家，中国驰名商标25件。全市市场主体54.3万户，增速连续三年全省第一。民营经济增加值完成2178.6亿元，年均增长8%。

（五）坚持不懈治理污染，生态环境持续改善。全力打好大气治理攻坚战，减煤、治企、控车、降尘、增绿“多管齐下”，取缔“三小”及重污染小企业5222家、实心粘土砖瓦窑486座，拆除烟囱2872根，淘汰改造燃煤锅炉3516台，完成48台燃煤发电机组超低排放改造，关停整治露天矿山53座，淘汰黄标车和老旧车33.3万辆，对京港澳高速公路穿城段、环城路过境货车实行限行分流，完成中心城区15.77万户“煤改气”、25.5万户焦炉煤气置换天然气，4家重污染企业搬迁改造取得实质性进展。2016年，全市单位生产总值能耗比2012年下降19.8%，煤炭消费量比2012年削减755.3万吨，空气质量综合指数、PM2.5平均浓度分别比2013年下降35.2%和40.6%，荣获省“十二五”节能减排工作先进市。山水林田湖生态修复整体推进，地下水超采综合治理工程实现压采能力4.8亿立方米，滏阳河、沁河、洺河退污还清加快实施，东武仕水库网箱养鱼全部取缔，累计造林绿化228.5万亩，全市森林覆盖率达29.2%，比2012年提高6个百分点，荣获全国绿化模范城市。

（六）加强基础设施建设，承载能力大幅跃升。邯黄铁路、邯长邯济铁路扩能改造竣工通车，铁路年集疏运能力由1.09亿吨提高到6.1亿吨。邯大高速、京港澳高速邯郸段改扩建、绕城高速东南环建成通车，太行山高速开工建设，实现县县通高速公路。机场二期扩建竣工，开通10条航线，民航吞吐量达46.3万人次，是2012年的2.7倍。邯郸国际陆港建成运营，邯郸海关挂牌开关。水利、能源设施不断完善，南水北调中线邯郸段建成通水，20个配套水厂全部投用，引黄入冀补淀工程邯郸段具备通水条件，基本实现县县通天然气。

（七）大力推进新型城镇化，城乡面貌显著改观。城镇规划体系日臻完善，城市布局更加优化。东区“八纵八横”路网初步形成，河北工程大学迁建、中央商务区、科技中心、青少年活动中心等重大工程开工在建，客运枢纽一期、东区水厂等公共服务设施竣工。老城区新建改建道路114条次、小街巷200条次，改造城中村和旧城区片94个，滏阳河主城区段游船通航，串城街北段改造主体完工，供水供热供气、垃圾污水处理等设施不断完善。城市精细化管理水平进一步提高，我市列入国家智慧城市试点，主城区公共区域实现WiFi全覆盖，连续五届入围“中国城市信息化50强”。县城建设步伐加快，建成6个国家级、8个省级园林县城，大名县列入省级历史文化名城。创建省级美丽乡村145个，馆陶县寿东村、邯山区小堤村荣获全国十大最美乡村。全市常住人口城镇化率达53.53%，比2012年提高7个百分点。

（八）致力保障改善民生，社会发展和谐稳定。财政用于民生支出累计达1272.4亿元，办成342件民生实事。城镇、农村居民人均可支配收入分别达26603元和12153元，年均增长8.9%和10.2%。扶贫开发取得显著成效，60.4万贫困人口稳定脱贫。城镇新增就业50.9万人，新增农村劳动力转移就业25万人。社保体系日益完善，城乡居民基本医疗保险实现整合、基本养老保险和大病保险实现全覆盖，城镇企业退休人员基本养老金连续12年上调，城乡低保标准分别比2012年提高32.1%和46.5%，农村互助幸福院、城市社区居家养老服务中心覆盖率分别达82%和65%。保障性安居工程竣工9.39万套，改造农村危房3.36万户。解决了275.9万农村人口饮水安全问题。教育教学质量和办学条件不断提高，17个县（市、区）通过国家义务教育发展基本均衡县评估认定，建成农村标准化学校618所，我市被确定为全国学前教育改革发展试验区。公共文化服务体系进一步完善，成功举办中原民间艺术节、中国吹歌节等品牌文化活动，推出了电视剧《太行山上》等一批精品力作，与央视合作成功举办《中国成语大会》总决赛，《邯郸市志》出版发行。医药卫生体制改革成效明显，公共医疗卫生和计划生育服务体系覆盖城乡，“全面两孩”政策落地实施，我市列入国家级公立医院综合改革试点城市和医养结合试点城市。全民健身运动蓬勃开展，成功举办两届国际太极拳运动大会、两届全国“划骑跑”铁人三项挑战赛。加强和创新社会治理，构建了“三网合一”治安防控体系，深入推进畅通邯郸建设，依法打击和处置非法集资，建立医患纠纷化解新模式，刑事立案总量比2012年下降57.4%，食品药品安全监管力度加大，安全生产形势总体平稳，信访工作得到加强。文明城市创建扎实推进，市民文明素质不断提升。国防动员和双拥共建深入开展，荣获全国双拥模范城“八连冠”、全国人民防空先进城市。民族宗教、新闻出版广电、外事侨务、邮政通信、防震减灾、援藏援疆、妇女儿童、档案、气象、老龄、残疾人等工作都取得了新成绩。

同时，我们更加注重加强政府自身建设，深入开展党的群众路线教育实践活动、“三严三实”专题教育和“两学一做”学习教育，认真落实中央八项规定精神，坚决纠正“四风”，加强机关作风建设，行政效率和服务质量进一步提高。积极推进依法行政，公布了市政府部门权力、责任、监管、收费、负面、中介服务六张清单，政府立法和重大行政决策制度更加完善，政府法律顾问制度实现市县乡全覆盖，审计监督和反腐倡廉工作力度不断加大。自觉接受人大、政协监督，认真办理人大代表建议、政协提案，按时办复率均为100%。

各位代表！2016年，是我市大事多、要事多、难事多的一年，全市人民齐心协力、共克时艰，顶住了压力、经受了考验、战胜了挑战。面对历史罕见的“7·19”特大洪灾，我们紧急动员、迅速行动，第一时间全力开展抗洪抢险救灾，转移安置群众17万余人，9天抢通路电讯，最大限度保护了人民群众生命财产安全，最大限度减少了灾害损失，灾后重建取得阶段性胜利。在这场生死考验中，邯郸儿女众志成城、共抗洪灾，展示出强大的凝聚力和战斗力。面对艰巨繁重的化解过剩产能任务，我们下定决心，严格标准，加强监管，提前超额完成全年任务，妥善安置分流职工，创造性建立了全国首个钢铁产能交易互助平台，经验得到推广。面对社会关注的部分行政区划调整工作，我们勇于担当、周密部署，一周时间顺利完成交接，平稳实现了区划调整，市区面积由655.78平方公里扩大到2661.83平方公里，为打造区域中心城市奠定了坚实基础，实现了全市人民多年来的夙愿。

各位代表！过去四年，我市在“三期叠加”、压能治污力度空前的背景下，取得上述成绩实属不易、难能可贵。这主要得益于省委、省政府和市委的正确领导，得益于市人大、市政协和社会各界的鼎力支持，凝聚着全市广大干部群众的智慧、心血和汗水。在此，我代表市人民政府，向全市人民，向人大代表、政协委员，向各民主党派、工商联、无党派人士和人民团体，向各位老领导、老同志，向驻邯人民解放军、武警官兵和政法干警，向中直、省直驻邯各单位，向所有关心支持邯郸发展的同志们、朋友们，致以崇高的敬意和衷心的感谢！

各位代表！在肯定成绩的同时，我们也清醒地认识到，发展不够好、不够快仍是邯郸面临的主要矛盾，经济社会发展中还存在不少困难和问题：一是发展质量效益还不高，传统产业比重较大，新兴产业发展不足，新旧动能转换不快，科技创新能力不强，经济外向度较低；二是生态治理和修复任务繁重，大气污染防治形势严峻，空气质量与群众期盼还有不小差距；三是城市承载能力不强，城市经济和县域经济发展不快，城镇化水平有待进一步提高；四是社会事业发展存在不少短板，公共服务供给能力不足，城乡居民收入不高，全面建成小康社会仍有很多薄弱环节；五是一些新的矛盾和问题凸显，社会稳定风险、金融领域风险加大；六是有的地方落实新发展理念不够自觉，市场营商环境亟待改善，一些部门和干部不作为、乱作为现象仍然存在。对此，我们一定高度重视不回避，勇于担当不懈怠，切实有效加以解决，决不辜负全市人民的厚望！

二、今后五年的发展目标和主要任务

未来五年，是我市加快转型发展至关紧要、非常关键的时期。刚刚闭幕的市第九次党代会，研判了邯郸所处的历史方位，分析了前进道路上的机遇和挑战，描绘了未来五年发展的宏伟蓝图，激发了全市人民干事创业的昂扬斗志。我们一定要抢抓机遇、乘势而上，坚定走好发展新路，谱写壮美绚丽的新篇章！

今后五年，政府工作的指导思想是：全面贯彻党的十八大和十八届三中、四中、五中、六中全会精神，以邓小平理论、“三个代表”重要思想、科学发展观为指导，深入学习贯彻习近平总书记系列重要讲话精神，统筹推进“五位一体”总体布局，协调推进“四个全面”战略布局，认真贯彻创新、协调、绿色、开放、共享的发展理念，大力推进供给侧结构性改革，认真落实省、市第九次党代会各项决策部署，继续解放思想、深化改革、扩大开放，坚定走加快转型、绿色发展、跨越提升新路，全面做好稳增长、促改革、调结构、治污染、惠民生、防风险各项工作，不断提升区域中心城市地位，为加快建设宜居宜业宜游的富强邯郸、美丽邯郸而努力奋斗。

按照市第九次党代会提出的“四超、一个全面建成”总体目标，今后五年，全市主要发展目标是：经济实力明显提升。全市生产总值达到5000亿元以上，人均生产总值达到5万元以上，全部财政收入达到500亿元以上，一般公共预算收入达到300亿元以上。结构调整明显加快。服务业在国民经济中的比重超过第二产业，装备制造业、高新技术产业占规模以上工业比重分别达到25%和20%以上。发展动能明显转换。重点领域改革取得重大突破，创新体系建设迈出重大步伐，科技进步贡献率提高10个百分点，迈进国家创新型城市试点行列。环境质量明显改善。主要污染物排放总量持续下降，PM2.5平均浓度降至55微克/立方米，全市平均优良天数争取达到260天以上，森林覆盖率稳定在35%，建成国家森林城市，主要河流水质达到水功能区划标准。文明程度明显提高。建成全国文明城市，社会治理体系现代化基本实现。城乡面貌显著改善，常住人口城镇化率达到60%以上。居民收入明显增加。城乡居民人均可支配收入年均增长快于经济增长，脱贫攻坚任务全面完成，全面小康成果得到巩固和提高。

实现上述目标，重点抓好以下五大任务：

（一）坚定不移调整产业结构，努力提高发展质量效益。精准把握供给侧结构性改革要求，不折不扣完成去产能任务，钢铁产能控制在4000万吨以下，煤炭产能控制在2300万吨以下。积极处置“僵尸企业”，妥善做好职工安置、资产处置和债务化解工作。深入实施《中国制造2025》和“互联网+”行动计划，推动钢铁、煤炭、焦化、建材等传统产业“瘦身强体”。加快发展先进装备制造、新材料、新能源汽车、生物、“大智移云”信息技术、数字创意、节能环保、储能、氢能等战略性新兴产业，推进军民融合产业发展，建设全国重要的先进制造业基地。突出抓好现代物流、文化旅游、金融保险、电子商务、会展经济、总部经济等现代服务业，服务业增加值占生产总值比重提高到45%以上，建设文化旅游强市、中国北方重要的商贸物流中心。到“十三五”末，将精品钢材、装备制造、食品工业、节能环保、新能源汽车、现代物流六大产业，打造成为超千亿元产业。扎实推进农业供给侧结构性改革，大力发展高效生态农业，推动粮经饲统筹、农林牧渔结合、种养加一体、一二三产业融合发展，延伸产业链、提升价值链，不断增加农民收入。

（二）坚定不移治理环境污染，努力建设绿色美丽家园。完善大气污染防治顶层设计，统筹实施控煤减排降尘措施，坚决打好蓝天保卫战；持续推进河湖水系综合治理，着力解决城市黑臭水体问题；认真抓好土壤和农业面源污染防治，确保大气、水和土壤环境“三年有好转、五年大改善”。加强重点地区、领域、行业、企业技术减排和污染治理，基本完成主城区4家重污染企业搬迁改造，开展县城工业污染源退城搬迁，加快调整能源结构、运输结构和出行结构，实施燃煤替代行动，加强扬尘和机动车尾气治理，大幅减少污染物排放总量。实施山水林田湖生态保护和修复工程，持续开展“绿美邯郸”攻坚行动，每年造林绿化52万亩以上；落实最严格的耕地保护、集约节约用地和资源管理制度，推进退耕还林和露天矿山环境治理；搞好重点生态功能区保护，加强地下水超采综合治理，持续提高生态支撑能力。

（三）坚定不移深化改革创新，努力增强发展动力活力。坚持以改革释放发展红利，深化行政管理体制改革，建立覆盖市县的行政审批局和规范的公共资源交易市场体系，全面优化市场营商环境。深入推进国有企业改革，稳妥推进农村改革，积极推进金融、财税、投融资等各领域改革，更好服务和支持实体经济发展。广泛参与京津冀、中原经济区分工协作，主动融入“一带一路”发展战略，深化与环渤海地区的产业合作，支持有实力的企业走出去，提高对外开放的广度和深度。加快实施创新驱动发展战略，大力发展科技型中小企业和高新技术企业，健全产学研协同创新机制，吸引更多的科技成果到我市孵化转化。推动大众创业、万众创新，建设一批“双创”基地，培育一批创新型企业家、技术专家和创客团队，打造创客“栖息地”和“聚集区”。

（四）坚定不移推动协同发展，努力打造区域中心城市。围绕建设京津冀世界级城市群中的区域中心城市目标，按照“三核驱动、组团发展”的要求，优化都市圈空间布局，完善城市功能，提升承载能力，全面加快东区、老城区和冀南新区建设步伐，提高中心城区首位度。坚持以交通圈拓展都市圈、带动经济圈、扩大辐射圈，加快实施太行山高速、城市轨道交通、京石邯城际、聊邯长客专、邯郸动车所等重大交通基础设施项目，建设全国综合交通枢纽城市。推进县城扩容提质升级，支持武安市建成邯郸市副中心，涉县、大名、魏县、曲周建成区域次中心城市，磁县、成安、临漳、馆陶、鸡泽、广平、邱县建成地域特色明显的城市，重点建设一批园区经济、休闲旅游、商贸物流、教育科技、传统文化等特色小镇，基本实现美丽乡村建设全覆盖，努力形成以中心城区为核心，副中心城市、区域次中心城市、特色县城、特色小镇梯次推进的城镇化格局。大力发展县域经济，培育壮大乡域经济，推动县域经济增比进位突破。建好冀南新区、邯郸经济技术开发区、冀津循环经济产业示范区等承接平台，积极承接京津产业转移和北京非首都功能疏解，加快建设京津先进制造业转移承接地、食品工业扩能首选地、优质医疗教育资源合作优选地和旅游目的地。

（五）坚定不移创新社会治理，努力提高人民生活水平。牢固树立以人民为中心的发展思想，切实解决好人民群众最关心、最直接、最现实的利益问题，让人民群众在共建共享中有更多获得感和幸福感。坚持精准扶贫、精准脱贫，确保到2020年贫困县全部摘帽、贫困村全部出列、贫困人口全部稳定脱贫。千方百计扩大就业，深化收入分配制度改革，提高低收入群体收入水平。实施社保扩面提标工程，均衡配置城乡教育资源，统筹推进城乡基础教育一体化发展，完善城乡医疗健康服务体系。培育和践行社会主义核心价值观，加快构建现代公共文化服务体系，加强历史文化遗址和非物质文化遗产保护利用，充分挖掘文化资源，打造文艺精品，讲好邯郸故事，推动文化产业成为支柱产业。深化平安邯郸建设，强化公共安全管理，提高食品药品安全监管水平，全面提升防灾减灾救灾能力，维护社会和谐稳定大局。

各位代表！走好发展新路，全面建成小康社会，是全市人民的共同意志和热切期盼。只要我们坚定信心、勇往直前，美好蓝图就一定能够变为现实，一个宜居宜业宜游的富强邯郸、美丽邯郸就一定能够呈现在世人面前！

三、2017年目标任务和重点工作

今年是党的十九大胜利召开之年，是供给侧结构性改革的深化之年，也是全面落实省、市第九次党代会精神的开局之年。做好今年工作，意义重大、影响深远。

经济社会发展的主要目标是：全市生产总值增长7%左右，其中规模以上工业增加值增长5.5%以上，服务业增加值增长9.5%以上；一般公共预算收入增长7%；社会消费品零售总额增长10%左右；实际利用外资增长5%；城镇、农村居民人均可支配收入分别增长7.5%和8%；居民消费价格涨幅控制在3%左右；城镇登记失业率控制在4.5%以内；PM2.5平均浓度下降12%左右；单位生产总值能耗下降率和化学需氧量、氨氮、二氧化硫、氮氧化物等主要污染物减排完成省定任务。

做好今年经济社会发展工作，我们必须牢固树立和全面践行创新、协调、绿色、开放、共享的发展理念，坚定走加快转型、绿色发展、跨越提升新路，突出把握好六个方面：一是必须坚持“稳中快进”的总基调。进一步增强责任意识和争先意识，加力加压加码，奋发有为，勇于进取，全面加快发展步伐，不断推动跨越赶超。二是必须以推进供给侧结构性改革为主线。保持战略定力，坚决去、主动调、加快转，全力抓好去产能、去库存、去杠杆、降成本、补短板任务，减少无效低效供给，扩大有效供给，实现向高水平供需平衡跃升。三是必须始终坚守生态底线。保护环境就是保护生产力，改善环境就是发展生产力，始终像保护眼睛一样保护生态环境，像对待生命一样对待生态环境，科学施策，标本兼治，坚定不移走生态优先、绿色发展之路。四是必须牢牢扭住项目建设这个“牛鼻子”。把项目作为邯郸发展的生命线，将经济社会发展的各项任务转化为一个个具体项目，不断扩大有效投资，靠优质增量稳增长、调结构、扩总量。五是必须依靠改革创新推动转型升级。进一步解放思想、敢闯敢试，努力在简政放权、优化环境、创新创业等领域打造亮点、取得实效，让改革更加精准地对接发展所需、基层所盼、民心所向，让创新更加有效地积累发展势能、释放发展潜能、激活发展动能。六是必须更加注重保障改善民生。坚持把更多的公共资源向民生薄弱环节倾斜，不断提高广大人民群众生活质量和水平，持之以恒为群众办实事、解难事，促进社会公平正义，把发展硬道理更多体现在增进人民福祉上。

重点做好八个方面的工作：

（一）着力加快新旧动能转换。坚持“调旧”“育新”协同发力，改造提升传统动能，着力发展壮大新动能，推动产业向中高端迈进。抓牢抓实重点项目建设。继续坚持项目建设“三集中”工作机制，进一步优化投资结构，引导资金更多投向战略性新兴产业、现代服务业等领域。实施市重点项目300个，完成年度投资783亿元。突出抓好33个省重点项目，完成年度投资200亿元以上。坚定不移化解过剩产能。坚决落实习近平总书记提出的“四个决不允许”要求，严格执行环保、能耗、水耗、质量、技术、安全六类标准，用好规划、土地、社保、税收四个“杠杠”，压减炼铁产能484万吨、炼钢产能354万吨、煤炭产能175万吨、焦炭产能320万吨，淘汰落后火电装机规模6.9万千瓦。继续实施援企稳岗政策，多渠道分流安置转岗人员，确保职工就业有出路、生活有保障。在去产能的同时，加快改造提升传统动能，实施一批技改项目，推动“两化”深度融合，让传统产业焕发新活力。积极推进钢铁企业整合重组，确保永洋特钢退城搬迁一期投产，开工建设太行、冀南钢铁退城进园项目，完成文安、新金钢铁退城进园和宝信钢铁整合进园项目手续办理工作。培育壮大战略性新兴产业。实施“115”工程，扶持100家高新技术企业，推进100个新兴产业项目，建好50家研发中心（实验室），重点抓好中国电子北方产业基地、中化精细化工产业园、中船重工718所特种气体、中钢研人造蓝宝石、武安新能源产业园四期、中原智能装备制造基础件产业基地等一批战略支撑项目，确保高新技术产业增加值占规模以上工业比重达到16%以上。做大做强现代服务业。推进华耀城、浙江商贸城、林安智慧物流、万合公铁港多式联运、美食林环球中心等商贸物流项目尽快竣工投用，支持武安保税物流中心、青岛保税港区邯郸（鸡泽）功能区建设运营，积极申建邯郸综合保税区，大力发展跨境电商。加快推进冀南（永年）快递园区建设，打造快递区域分拨仓储中心。实施旅游产业化战略，推动全域旅游和产业融合发展，支持涉县、峰峰办好旅游产业发展大会，加快武安东太行、磁县溢泉湖、广府太极文化产业园、紫山景区等旅游项目建设，完善配套设施，推动更多的旅游景区提档升级，中华成语文化博览园年内竣工开放，全市旅游业总收入增长25%以上。加快发展文化、体育、健康、养老等幸福产业，扩大数字家庭、在线教育等信息消费，促进电商、快递进社区进农村，释放潜在消费需求。大力振兴实体经济。开展增品种、提品质、创品牌“三品”专项行动，发扬“工匠精神”，培育更多的“百年老店”，提高企业核心竞争力。深入开展帮扶企业活动，取消规范一批涉企收费项目，进一步降低制度交易和企业用能、用工、物流等环节成本。积极推进“个转企、小升规、规改股、股上市”，年内新增入统工业企业100家、挂牌上市企业50家。进一步疏通金融服务实体经济的“管道”，引导金融机构把资金更多投向实体经济，有效缓解中小微企业融资难、融资贵问题。加快推进科技创新。新增科技型中小企业1200家、科技小巨人企业60家、高新技术企业60家。加快培育创新载体，推动20%以上的规模以上企业建立研发机构，支持晨光集团建设国家级工程技术研究中心。加速科技成果转化，争创国家知识产权示范培育城市。实施人才强市战略，注重智库建设，实行更具灵活性和吸引力的人才政策，积极引进海内外科技领军人才、创新团队、管理人才和高技能人才，广聚天下英才而用之。充分激发“双创”活力。大力推广众创、众包、众扶、众筹商业模式，支持开发区、高校、企业盘活存量设施和场地，使各类主体各展其长、线上线下良性互动，建设市级以上众创空间40个。支持分享经济发展，提高资源利用效率，让更多的人参与进来、富裕起来。

（二）着力推动协同发展。坚持深度融入京津冀协同发展战略，全面加强与京津对接合作，唱好协同发展“重头戏”。推进协同创新。用足用好京津创新资源，深化与京津高等院校、科研院所的合作，深入推进以市校、县校、企校为重点的校地合作，争取3—5家知名高校、国家实验室和一批重大科技专项、重点科研项目落户邯郸。加快北京大学邯郸创新研究院发展，启动北京大学邯郸科技园建设，打造京津冀协同创新的典范。同时，加强交通、环保、教育、医疗、旅游、养老、金融等多领域合作，争取更多的京津名校、医院在我市设立分支机构。做强承接平台。加快开发区改革创新，深入推进机构编制、人事薪酬、行政审批、投融资等改革，进一步完善基础设施和配套服务设施，培育壮大特色产业集群，切实把开发区打造成转型升级示范区、对外开放主战场。支持有条件的县（市、区）创建省级高新区，支持复兴区建设省级经济开发区，省级以上开发区主要指标增速不低于20%。提高开放层次。围绕“215”目标，持续加大招商引资力度，发扬“四千四万”精神，以京津、长三角、珠三角等区域为重点，加强与央企、国内外知名企业的对接合作，积极开展走进央企及京津企业走进邯郸行动，引进一批品牌、市场、技术成熟的大项目、好项目。冀南新区、邯郸经济技术开发区各引进1个超百亿元的战略支撑项目，其他县（市、区）争取各引进1个超五十亿元的重大产业项目。落实和完善鼓励出口政策，抓好出口基地建设，促进外贸出口回稳向好。

（三）着力改善生态环境。坚持绿水青山就是金山银山，持续加强生态环境治理，让绿色成为邯郸的永久底色。实施“蓝天行动”。全面落实省委、省政府《关于强力推进大气污染综合治理的意见》，突出科学治霾、精准治霾、协同治霾、铁腕治霾，以壮士断腕的决心、刮骨疗毒的勇气、凤凰涅槃的气魄，扎实开展散煤治理、工业达标排放、扬尘综合整治等18项专项攻坚治理。上半年，将6大类45项“小散乱污”企业清零整治到位。全市完成“煤改气”“煤改电”10万户以上，抓好精准减煤、清洁高效集中利用和农村散煤替代等重点工作，实现中心城区散煤清零目标，全年削减煤炭消费300万吨。强化机动车尾气治理和燃油品质管理，提高黄标车拆解率，大力推广应用新能源汽车。加快邯郸热电厂搬迁前期工作，实施邯钢东区焦化厂搬迁，推进纵横钢铁关停转型。积极对标德龙钢铁、旭阳焦化，完成钢铁行业治污设施升级改造，焦化行业全部安装脱硝设施。科学实施采暖季工业企业错峰生产，坚决落实重污染天气应急预案，严格执行大气污染物特别排放限值要求。落实好建筑施工和道路扬尘控制措施，严禁露天焚烧。实施“网格化”管理，做到信息化、标准化、责任化、规范化，努力实现管理无死角、监督无盲区。严格环境执法和督查问责，严厉打击偷排偷放行为。实施“碧水行动”。实行市县乡三级河长制，统筹地表水、地下水环境治理，完成县城以上集中式饮用水水源地安全防护。落实省十大重点行业清洁化改造、城市黑臭水体治理、入河排污口污染整治三大专项行动，完成沁河改造清水入沁工程，全面消除中心城区、县城建成区黑臭水体。完善河流跨界断面生态补偿机制，以奖惩问责倒逼责任落实。实施“净土行动”。出台土壤污染防治计划实施细则，开展土壤污染治理与修复试点，抓好工业大宗固体废物和尾矿资源化利用，集中治理受污染农用地。搞好山水林田湖生态综合修复，推进地下水超采综合治理，提高南水北调引江水使用比例和效益，治理水土流失面积150平方公里，完成造林绿化66万亩。保护环境人人有责，要全市动员、全民参与，打好大气污染综合治理的人民战争。只要我们持续用力、久久为功，就一定能享受到更多的蓝天白云、碧水青山。

（四）着力推进农业供给侧结构性改革。坚持以增加农民收入、保障有效供给为主要目标，以提高农业供给质量为主攻方向，以体制改革和机制创新为根本途径，优化农业产业体系、生产体系、经营体系，促进农业增效、农民增收、农村增绿。大力发展高效生态农业。把增加绿色优质农产品供给放在突出位置，加快构建粮经饲协调发展的三元结构，提高农业农村综合效益和竞争力。坚持质量兴农，狠抓农产品质量安全监管和品牌创建。发展设施农业，做优做强畜牧、蔬菜、果品产业，推进生态型标准化示范基地建设。以邯郸国家农业科技园区为引领，高水平打造3个示范型现代农业产业园区，新建15家市级以上现代农业园区、2家省级农业科技园区。推进高标准农田和农田水利基本建设，发展节水农业。培育壮大新产业新业态。加快发展现代食品产业，抓好曲周首农有机食品等100个农产品加工项目，培育壮大一批行业领军型龙头企业，创建10家省级、30家市级农业产业化示范联合体，农业产业化经营率提高1个百分点。推进“互联网+”现代农业行动，推动农业全产业链改造升级，积极发展农村电商、休闲农业、乡村旅游等新业态，促进农村一二三产业融合发展，培育农业农村发展新动能。深化农村重点领域改革。落实农村土地“三权分置”办法，发展土地托管、土地股份合作、订单农业等多种形式适度规模经营。基本完成农村土地承包经营权确权登记颁证，推进农村集体产权制度改革，争取实现县级农村产权交易市场全覆盖。

（五）着力拓展改革深度广度。坚持上下联动、精准发力，聚焦构建发展新体制，推动各项改革举措落地生根、见到实效。推进关键领域改革。深化国有企业改革，积极发展混合所有制经济，扎实推进企业破产改制及职工安置工作，妥善解决破产企业历史遗留问题，切实做好“三供一业”分离移交工作。深化投融资体制改革，加快融资平台市场化转型，大力推广政府和社会资本合作（PPP）模式，放大财政资金作用，撬动金融保险资本和社会资本。深化金融改革，支持邯郸银行做大做强，完成6家农村信用社改制，引进更多的域外金融机构入驻我市。推进企业资产证券化，加大股权融资力度，降低企业杠杆率。加强金融和政府债务风险防控，建立健全社会信用体系，构建地方金融监管体系，依法严厉打击恶意逃废银行债务、非法集资行为，优化金融生态环境，坚决守住不发生系统性和区域性风险底线。抓好医保、生育保险合并改革试点工作，有序推进事业单位分类改革和公车制度改革，深入推进城市公立医院综合改革、供销社改革等各项改革。优化市场营商环境。深化“放管服”改革，县（市、区）全部组建行政审批局，推进“互联网+政务服务”，进一步精简办事程序、缩短办理时限，取消各种不必要的证明和手续，让信息多跑路、企业少跑腿，全面提高服务效能。推进商事制度改革，实现“双随机、一公开”监管全覆盖，增强事中事后监管的有效性。切实发挥企业110和群众服务热线作用，对重点项目全程帮办代办，为企业排忧解难。加强政务诚信建设，严格履行各项约定义务，决不能“新官不理旧账”。严肃查处干扰、阻挠、破坏发展环境和市场经济秩序行为，努力打造政策洼地、服务高地、创业宝地。大力发展民营经济。认真落实各级扶持民营企业发展的政策措施，积极构建“亲”“清”新型政商关系，进一步拓宽市场准入，消除隐性壁垒，真心实意帮助企业解决好资金、信息、人才、市场等方面的实际困难。关心爱护企业家，搞好企业家培训，推动民营企业科技创新、调整结构，激发民营经济的活力和创造力。

（六）着力统筹城乡发展。坚持城市建设、城市管理、城市经济“三城齐抓”，生产、生活、生态“三生融合”，大力推进以人为核心的新型城镇化，让人民在城市生活得更美好。全市常住人口城镇化率提高1.7个百分点左右。强化规划引领作用。以行政区划调整为契机，坚持组团式、功能型、智慧化发展，进一步优化重大生产力和基础设施布局，统筹新设区与中心城区一体化发展，完成五期总体规划修编，构建现代化大都市格局。完善各项专项规划，抓好“多规合一”试点。提升城市承载能力。着眼把东区打造成为创新引领中心，以中央商务区为重点，推进东区综合路网、地下综合管廊、商贸地产等项目建设，开工建设市广电中心、客运枢纽二期等公共服务设施，市科技中心、市青少年活动中心建成投用。加快推进华夏幸福丛台产业新城建设。改造提升老城区，串城街北段文化旅游步行街竣工投用、修缮整治南段历史街区，打造邯郸文化新地标。建设改造陵西大街、东柳大街、北仓路等29条道路，打通滏东大街北延、建设大街北延、果园路等17条“断头路”，加快推进邯峰长输供水管线升级工程。实施新开河、支漳河整治、邯临沟改扩建等工程，增强城市防洪排涝能力。推进海绵城市建设，使城市既有“面子”、更有“里子”。建设一批重大工程。开工建设邯郸机场三期、主城区大外环、邯峰邯永邯肥三条快速路、107国道跨漳河特大桥、西部旅游路网等十大工程，邯郸火车站改扩建等项目竣工投用。提高精细化管理水平。进一步理顺城市管理体制，以治脏、治乱、治违为重点，在路平、街净、灯亮上下功夫，开展洁净城市、卫生城市、文明城市和智慧城市“四城同创”，创建一批“美丽城中村”和“美丽小区”，打造干净整洁、舒适宜居的城市环境。在主城区规划设置一批智能停车泊位，努力解决停车难问题。促进县城扩容提质。以公共设施补短板、产城教融合化、环境容貌整洁化、建设改造精品化为重点，进一步完善功能、提升品位、做大规模，推进乡改镇、村改居和户籍制度改革。不断提升公共服务水平，县以上城市主城区公共区域实现免费WiFi全覆盖。支持涉县开展国家新型城镇化综合试点建设。努力化解房产库存。坚持住房的居住属性，推动新“房九条”政策落地，严格住房市场监管，加大房地产中介整治力度，积极解决房地产遗留问题，促进房地产业平稳健康发展。大力推广装配式和绿色建筑，加快建筑业发展。推进美丽乡村建设。坚持“3+X”思路，丰富业态，连片打造，结合历史文化名镇名村和传统村落保护开发，每个县（市、区）建设1个美丽乡村重点片区，抓好峰峰响堂山、魏县梨乡水城2个省级片区，建设1000个美丽乡村重点村。

（七）着力打好脱贫攻坚战。坚持以“绣花”功夫精准扶贫、精准脱贫，做到“小康路上一个都不能少”，确保7.5万贫困人口稳定脱贫。多措并举力促贫困群众增收。实施特色农业、光伏、旅游、家庭手工业、电商、就业六大扶贫工程，实现贫困村特色扶贫产业和贫困户增收项目全覆盖，确保贫困人口收入稳定增长。推进订单培训、定向输出，转移贫困劳动力4万人以上。鼓励贫困家庭以到户扶贫资金、土地承包经营权、生产资料等入股农民合作社或农业企业，力争覆盖70%以上的贫困户。全面落实兜底保障政策。重点抓好农村危房改造和魏县、大名、涉县44个村的易地扶贫搬迁，加强基本医疗保险、大病保险、医疗救助等医疗保障，落实国家扶贫开发重点县农村义务教育学生营养改善计划和“三免一助”等政策，实现符合条件贫困家庭全覆盖，确保实现“两不愁、三保障”。将符合条件的农村建档立卡贫困人口，全部纳入低保范围。着力补齐贫困村路水电讯、垃圾处理和排水等基础设施短板，争取10个贫困村建成省级美丽乡村。完善脱贫攻坚长效机制。继续深入推进驻村帮扶、“一帮一”结对帮扶，推广“政银企户保”金融扶贫模式，各类扶贫贷款规模达到20亿元以上。强化脱贫考核监督，严格脱贫退出机制，提高脱贫质量，坚决防止假脱贫、“被脱贫”、数字脱贫，确保脱贫得到群众认可、经得起历史检验。

（八）着力增进人民群众福祉。坚持民生是最大的政治，持续加大民生投入，不断提高民生保障和服务供给能力，使发展成果更多地惠及全市人民。继续办好民生实事。重点实施10个方面、82件民生实事。新增城镇就业10万人，下岗失业人员再就业2.97万人。进一步提高退休人员基本养老金，确保按时足额发放。新建30个精品农村幸福院、10个社区居家养老服务中心。实施城市棚户区改造10207套、基本建成3484套。抓好农村饮水安全巩固提升工程，确保79万人稳定受益。全面发展社会事业。优先发展教育事业，支持本地高校发展，启动邯郸大学城、职教城建设，加快建设市区西部省级示范性高中，持续改善薄弱学校办学条件，全市改扩建规范化幼儿园60所、标准化中小学35所，办好公平优质教育，进一步提高教育教学质量。全力打造健康邯郸，开展医养结合服务，推进家庭医生签约，促进分级诊疗，创建健康家庭、健康社区。积极发展文化事业和文化产业，实施中华优秀传统文化传承发展工程，加强文物安全保护，加快推进“一校三团”搬迁工作，提高基本公共文化服务均等化水平。广泛开展全民健身运动，统筹群众体育、竞技体育和体育产业协调发展。加快推进灾后重建。全部完成民房、水利、交通、医疗等9大领域15个方面重建任务，努力使灾区群众生产生活条件超过灾前水平。抓好重大水利设施、河道及河堤修复整治、山洪地质灾害防治等工程建设，增强抵御自然灾害综合防范能力。推动社会治理创新。高度重视信访工作，及时就地解决群众合理诉求，依法打击非访、闹访、缠访。推进安全生产领域改革发展，严格落实领导责任、部门监管责任和企业主体责任，重点抓好煤矿及非煤矿山、建筑施工、冶金、危险化学品等高危行业和领域专项整治，加强道路交通安全和消防安全管理，坚决遏制重特大事故发生。深入实施食品药品安全放心工程，坚持最严谨的标准、最严格的监管、最严厉的处罚和最严肃的问责，确保人民群众“舌尖上的安全”。进一步完善立体化、信息化社会治安防控体系，严厉打击各类违法犯罪行为，着力营造安定和谐的社会环境。加强国防教育和国防后备力量建设，巩固军政军民团结的好局面。统筹做好新闻出版广电、气象防震减灾、民族宗教、外事侨务、邮政通信、人民防空、史志档案、援藏援疆、扶老助残、妇女儿童等各项工作。

四、加强政府自身建设

新形势、新任务，对政府工作提出了新要求。全市政府系统要切实增强政治意识、大局意识、核心意识、看齐意识，坚决维护以习近平同志为核心的党中央权威，自觉向党中央看齐，全面加强政府自身建设，努力建设创新政府、高效政府、实干政府、法治政府和廉洁政府。

（一）提升学习力，建设创新政府。把学习作为一种政治责任和工作方式，加快知识更新，优化知识结构，拓宽眼界视野，在“学思践悟”中提升履职能力。坚持用改革思维、创新思维、开放思维、市场思维解决问题、推进工作，敢于突破常规，善于抓重点、破难点、有特点、争试点、打造亮点、当好示范点，以精道工作实现新突破、展现新作为。

（二）提升统筹力，建设高效政府。把统筹兼顾作为重要工作方法，牢固树立全市“一盘棋”观念，加强全局统筹、政策统筹、资源统筹、力量统筹，推行扁平化、信息化、精细化管理，推进治理体系和治理能力现代化，促进行政效率大提升，努力打造统筹有力、运转高效的政府。

（三）提升执行力，建设实干政府。把担当实干作为自觉追求，坚持干字当头，建立“四个干”抓落实机制，每项工作都明确干什么、怎么干、谁来干、什么时间干成，精准量化目标，精准明责问效，精准督导考核。坚持马上就办、办就办好，殚精竭虑、夙夜在公，事争一流、唯旗是夺，以奔跑的状态真抓实干，用实干实政推动发展、赢得未来。

（四）提升公信力，建设法治政府。把遵守宪法和法律作为施政的根本原则，坚持法定职责必须为、法无授权不可为，真正做到尊法学法守法用法。加强政府行政立法，做好行政复议和行政应诉工作，推行执法“三项制度”。健全依法科学民主决策机制，自觉接受人大法律监督和工作监督、政协民主监督，认真办理人大代表建议和政协提案，主动接受社会和舆论监督。加大政务公开和信息公开力度，将政府运行置于阳光之下。

（五）提升约束力，建设廉洁政府。把纪律和规矩挺在前面，认真落实全面从严治党要求，严格执行《准则》和《条例》。深入推进政府系统党风廉政建设，落实“两个责任”，强化“一岗双责”。坚决落实中央八项规定精神，深化机关作风整顿，一以贯之纠正“四风”。市县政府要带头过紧日子，一律按不低于5%的幅度压减一般性支出，坚守节用裕民的正道。加大行政监察和审计监督力度，严肃查处各种不正之风和腐败问题，努力实现干部清正、政府清廉、政治清明。

各位代表！雄关漫道真如铁，而今迈步从头越。站在新的历史起点上，让我们更加紧密地团结在以习近平同志为核心的党中央周围，在省委、省政府和中共邯郸市委的坚强领导下，凝心聚力，奋发进取，在加快转型、绿色发展、跨越提升的新征程上迈出铿锵步伐，为全面建成小康社会、加快建设宜居宜业宜游的富强邯郸美丽邯郸而努力奋斗，以优异成绩迎接党的十九大胜利召开！
